# Involvement of GATA1 and Sp3 in the activation of the murine STING gene promoter in NIH3T3 cells

**DOI:** 10.1038/s41598-017-02242-w

**Published:** 2017-05-18

**Authors:** Yan-Yan Xu, Rui Jin, Guo-Ping Zhou, Hua-Guo Xu

**Affiliations:** 1 Department of Laboratory Medicine, The First Affiliated Hospital, Nanjing Medical University, Nanjing, Jiangsu Province 210029 China; 2 Department of Pediatrics, The First Affiliated Hospital, Nanjing Medical University, Nanjing, Jiangsu Province 210029 China

## Abstract

Stimulator of Interferon Gene (STING) is a key mediator of innate immune signaling. STING plays a pivotal role in the pathogenesis of many diseases including infectious diseases, auto-immune diseases and cancer. Many studies have been carried out recently in the field of STING-regulated pathway, however, rarely of transcriptional mechanisms. To characterize the murine STING (mSTING) promoter, we cloned a series of different nucleotide sequences of the 5′-flanking region of the mSTING gene. Transient transfection of promoter-reporter recombinant plasmids and luciferase assay illustrated the region (−77/+177) relative to the transcription start site (TSS) of the mSTING gene was sufficient for full promoter activity. This region contains GATA1, IK2, Sp1/Sp3 and STAT putative transcription factor binding sites. Mutation of GATA1 or Sp1/Sp3 sites led to obvious decrease of the mSTING promoter activity. Overexpression of GATA1 and Sp3 enhanced the mSTING promoter activity, whereas knockdown of GATA1 and Sp3 by a siRNA strategy significantly reduced the transcription activity. Chromatin immunoprecipitation assays demonstrated that GATA1 and Sp3 interact with the mSTING promoter *in vivo*. These results provided the first analysis of mSTING promoter and demonstrated that transcription factor GATA1 and Sp3 positively regulate the basal transcription of the mSTING gene.

## Introduction

The innate immune system is the first line of defense against invading pathogens, which is initiated by different germline-encoded pattern recognition receptors (PRRs), such as Toll-like receptors (TLRs), Nod-like receptors (NLRs) and RIG-I-like receptors (RLRs)^1^. During infection, nucleic acids from invading pathogens are recognized by TLRs and RLHs, which then elicit a series of signaling events leading to the production of pro-inflammatory cytokines, type I interferons (IFNs) and chemokines^2, 3^.

Intracellular nucleic acid especially DNA is an important pathogen-associated molecular patterns (PAMPs) during infection with viruses, intracellular bacteria and protozoan. Many cytosolic DNA sensors acted as PPRs, such as IFI16, DDX11, Mre11, DNA-PK, cGAS, were discovered in the past decades^4–8^. In 2008, the discovery of a new sensor, named as STING, helped to understand the signaling pathway of immune-stimulatory DNA to mobilize immune responses^9^. STING, also known as MITA, ERIS, MPYS, TMEM173, is expressed ubiquitously in multiple human tissues and cell lines^9–13^. Recent researches showed that STING plays a key role in response to viral and bacterial pathogens, to self-DNA during autoimmune disease, and in DNA adjuvancy^14–16^. Once DNA is accumulated in cytoplasm, STING is activated by interaction with upstream DNA sensors and leads to binding and phosphorylation of TANK binding kinase 1 (TBK1). The STING-TBK1 complex phosphorylates IRF3, then translocated to the nucleus to induce transcription of type I interferon (IFN), as well as multiple interferon-stimulated genes (ISG)^17^. Unexpectedly, a recent study showed that STING can interact with conserved hemagglutinin fusion peptide (FP) of influenza A virus (IAV), a kind of RNA viruses, to evoke innate immune response^18^. What’s more, up to the present, many studies have linked STING to many physiological and pathological responses involving innate and adaptive immunity, and placed this innate immune adaptor at the center of both protective and detrimental processes *in vivo* ^19^. In the anti-tumor immunity, a protective role for STING during cancer immunotherapy has been revealed in mouse tumor models. STING in dendritic cells is activated by the recognition of tumor cell DNA, leading to IFN-β induction and adaptive immunity^20, 21^. However, the STING-signaling is generally suppressed in cancers, for example, in colorectal carcinoma. Loss of STING signaling impedes DNA damage response accountable for generating key cytokines that facilitate tissue repair and anti-tumor-T cell priming^22^. Elevation of the expression and activity of STING improve the anti-tumor effect in response to standard therapies and immunotherapies. In addition, other studies have linked STING to MHC class II signaling. That MHC class II complexes mediate cell death signaling required STING-dependent activation of the extracellular signal-regulated kinase signaling pathway^12^.

The STING homologues exhibit high identity in many species, such as Homo sapiens, Mus musculus, Bos Taurus and Susscrofa. STING gene encodes a protein of 379 amino acid (aa) in human and 378 aa in mouse and shares 81% similarity and 68% identity between human and murine^9^. Many studies have focused on the field of STING signaling pathway in the past several years, however, rarely of its transcriptional mechanisms. Here we first cloned the murine STING gene promoter region and investigated the molecular mechanisms involved in the mSTING transcriptional activity. The results showed that the proximal promoter of mSTING is located within the region −77/+177 nt relative to TSS and transcription factor GATA1 and Sp3 positively regulate the mSTING promoter activity.

## Results

### Bioinformatics analysis of 5′ regulatory region in mSTING gene

**Figure 1:**
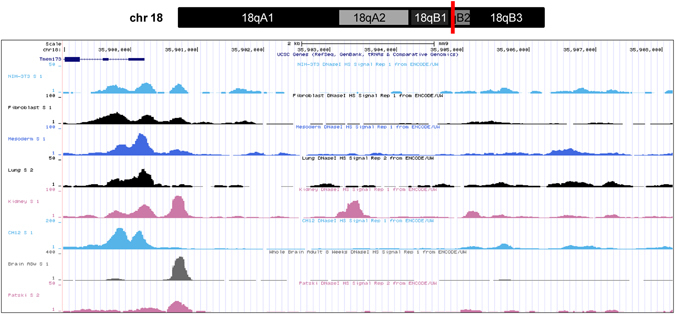
DNase I hypersensitive sites in 5′ upstream of the mSTING gene by ENCODE. Part of the chromosome 18 structure was shown on the top of diagram and the mSTING gene (NC_000084.6) location was marked by red line. The 5′-flanking region of the mSTING gene (chr18:35, 899, 540–35, 904, 129, 4590 nt) was analyzed for DNase I hypersensitive sites in several murine cell lines and tissues including NIH3T3 cells by ENCODE (http://genome.ucsc.edu/). As the bottom of figure shown by UCSC Genome Browser on Mouse July 2007 (NCBI37/mm9) Assembly, the peaks of DHS appeared around TSS of the mSTING gene.

Based on the NCBI database, sequence alignment was applied for the mSTING gene to identify the full sequence of the STING gene(chr18:35733678~35740554) and promoter prediction. Based on Encyclopedia of DNA Element (ENCODE) at UCSC (http://genome.ucsc,edu/), the region around TSS showed highly affinity of DNase I hypersensitive sites in several murine cell lines and tissues (Fig. 1). As many previous studies confirmed, the major regulatory elements usually exist in the proximal portion of 5′-flanking region of the gene, particularly within 1000 nt upstream from the translation start codon. As prediction of UCSC/ENCODE, there are some regulatory elements of 1000 nt upstream from TSS of mSTING in NIH3T3 and several other murine cell lines. Therefore, we identified the sequence from −828 to +177 nt (related to TSS) of 5′ upstream as a potential promoter.

### Cloning and promoter analysis of the mSTING 5′-flanking sequence

**Figure 2:**
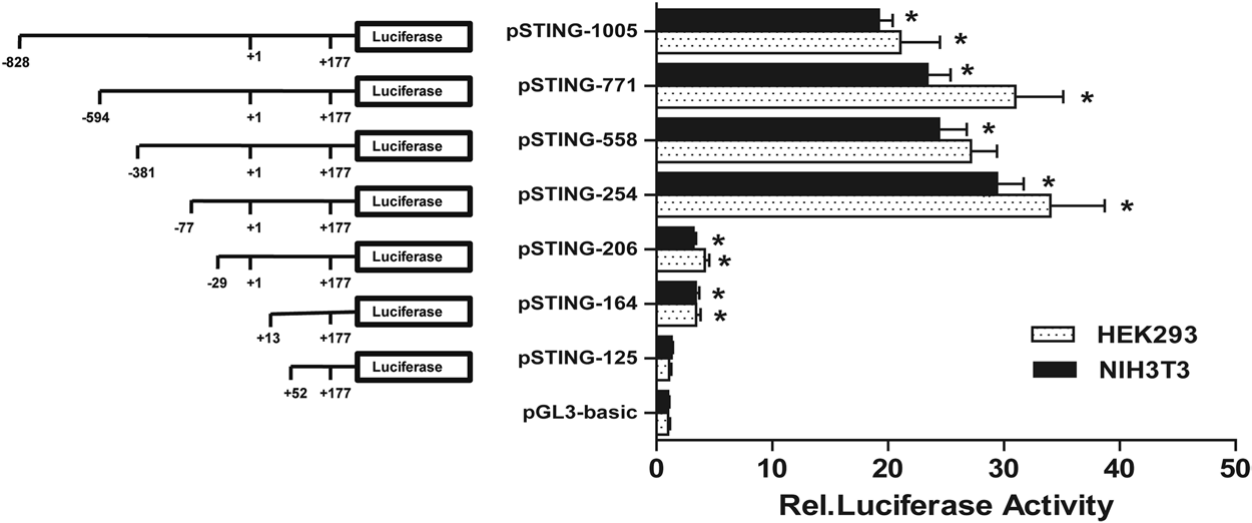
Deletion analysis of the mSTING promoter in NIH3T3 and HEK293 cells. Schematic structures of the mSTING promoter reporter constructs were shown on the left. The numbering was relative to the transcription start site. Seven recombinant reporter plasmids and pGL-3 Basic were cotransfected with the pRL-TK into NIH3T3 and HEK293 cells, respectively. The levels of firefly luciferase activity were normalized to the Renilla luciferase activity. Each bar represented the mean ± SD of three independent experiments (n = 3, *p < 0.05 vs. pGL3-Basic).

To identify the promoter of the mSTING gene, we cloned the fragment with the length of 1005 nt located at position −828 to +177 nt relative to the TSS into the promoterless pGL3-Basic luciferase reporter, named pSTING-1005. As shown in Fig. 2, luciferase assays revealed a 19.2-fold increase in promoter activity of pSTING-1005 compared to pGL3-Basic in NIH3T3 cells, indicating a functional promoter is contained in the region of −828~+177 nt of the mSTING gene. To determine the minimal sequence for regulating mSTING gene expression, a series of luciferase reporter plasmids containing different DNA fragments (gradually truncated 5′-end with the predicted promoter sequences of the mSTING gene) were generated, named as pSTING-771, pSTING-558, pSTING-254, pSTING-206, pSTING-164, and pSTING-125, respectively. These recombinant plasmids, along with pRL-TK were transiently transfected into NIH3T3 and HEK293 cells, and promoter activity was measured by luciferase activity assays. As shown in Fig. 2, deletion fragment from −77 to −29 nt relative to TSS resulted in a significant decrement of the transcriptional activity by 90%, and pSTING-125 had no difference in luciferase activity compared with pGL3-Basic in NIH3T3 cells. The same tendency could be observed in HEK293 cells. These results indicated that the sequence spanning nucleotides from −77 to +177 is sufficient for eliciting basal transcriptional activity of the mSTING gene.

### Mutational analysis of the mSTING promoter region

**Figure 3:**
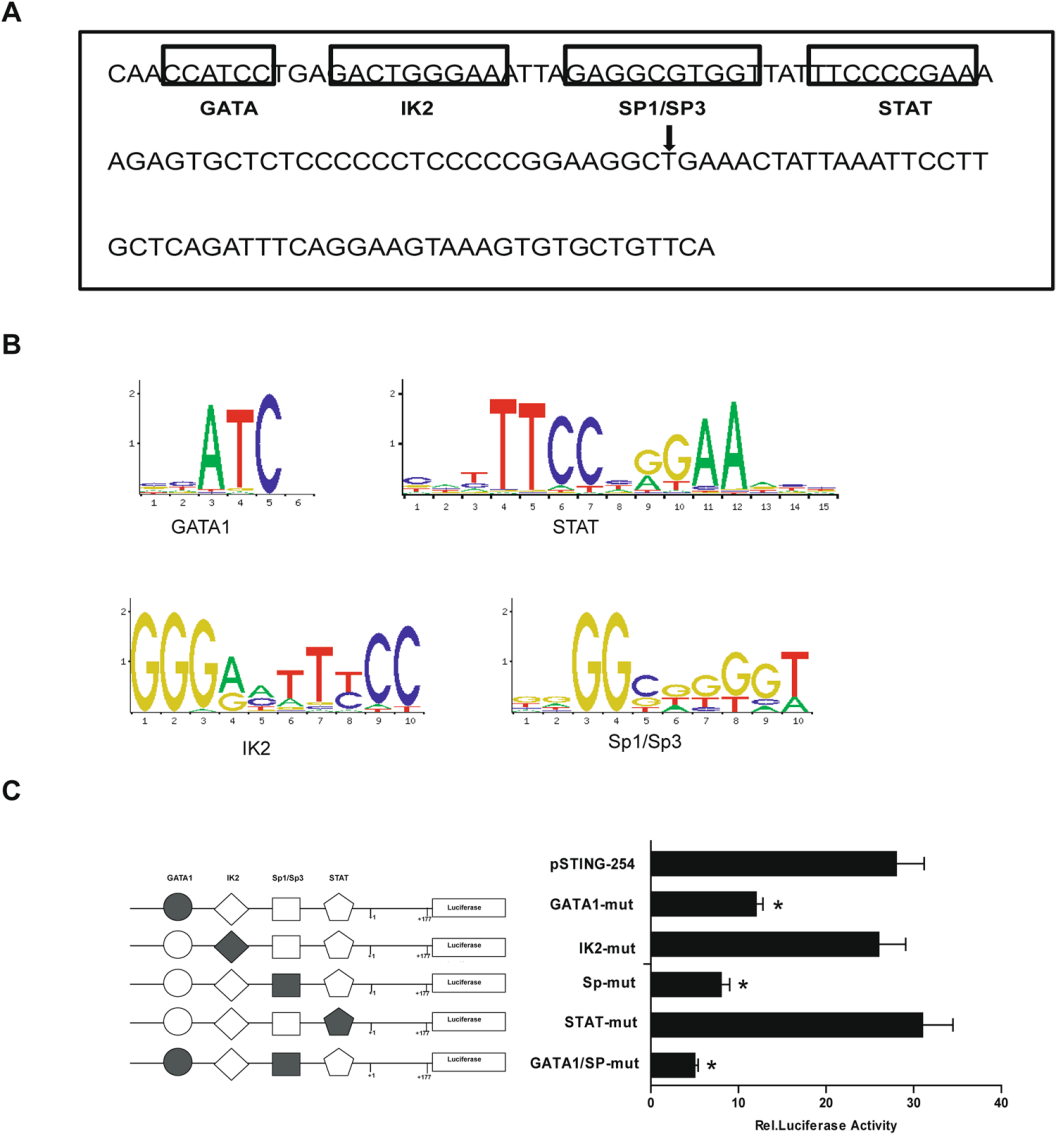
Prediction of transcription factor binding sites and mutation analysis of the mSTING minimal promoter. (**A**) A schematic representation of the putative binding sites for DNA-binding proteins in the proximal promoter of the mSTING gene. The 129 nt DNA sequence (from −77 to +52) was analyzed with TFSEARCH softwares. The putative transcription factor binding sites were outlined with black box, and the names of the transcription were annotated. The transcription start site (TSS) was marked by black arrow. (**B**) Conserved base sequence of GATA1, IK2, Sp1/Sp3 or STAT binding site. The base size presented the binding affinity coefficient of transcription factor and promoter. (**C**) Mutation analysis of the mSTING minimal promoter. The left: Binding sites for GATA1, IK2, Sp1/Sp3 and STAT were indicated with open different shapes. Mutations were shown in bold above the histogram. The right: The relative luciferase activities derived from mutational pSTING-254. The site-special mutagenized plasmids were cotransfected with pRL-TK into NIH3T3 cells and lucifarase assays were performed. The level of firefly luciferase activities was normalized to the Renilla luciferase activity. Each bar represented the mean ± SD of three independent experiments. (n = 3, *p < 0.05 vs. pSTING-254).

Using online software TFSEARCH ver.1.3., we found several putative binding sites, including GATA1, IK2, Sp1/Sp3 and STAT in the promoter region of mSTING (Fig. 3A). To determine the functions of these putative binding sites, the key nucleotides of TFBSs were predicated by JASPAR database (Fig. 3B) and site-direct mutagenesis were carried out based on the wild-type pSTING-254. We transfected these mutational plasmids into NIH3T3 cells and measured the luciferase activities 24 h later. As shown in Fig. 3C, compared with the wild-type pSTING-254, mutation of the IK2 or STAT binding site had no effect on the promoter activity of mSTING. In contrast, mutations of the GATA1 or Sp1/Sp3 binding site caused dramatically decrease in mSTING promoter activity. Moreover, mutation of both GATA1 and Sp1/Sp3 binding site led to less promoter activity compared with the sole binding site mutation. Because the GATA1 and Sp1/Sp3 site contained no overlapping DNA-binding domains, these results suggested that both GATA1 and Sp1/Sp3 are essential for basal transcription of the mSTING gene.

### GATA1 is involved in activation of the mSTING promoter

**Figure 4:**
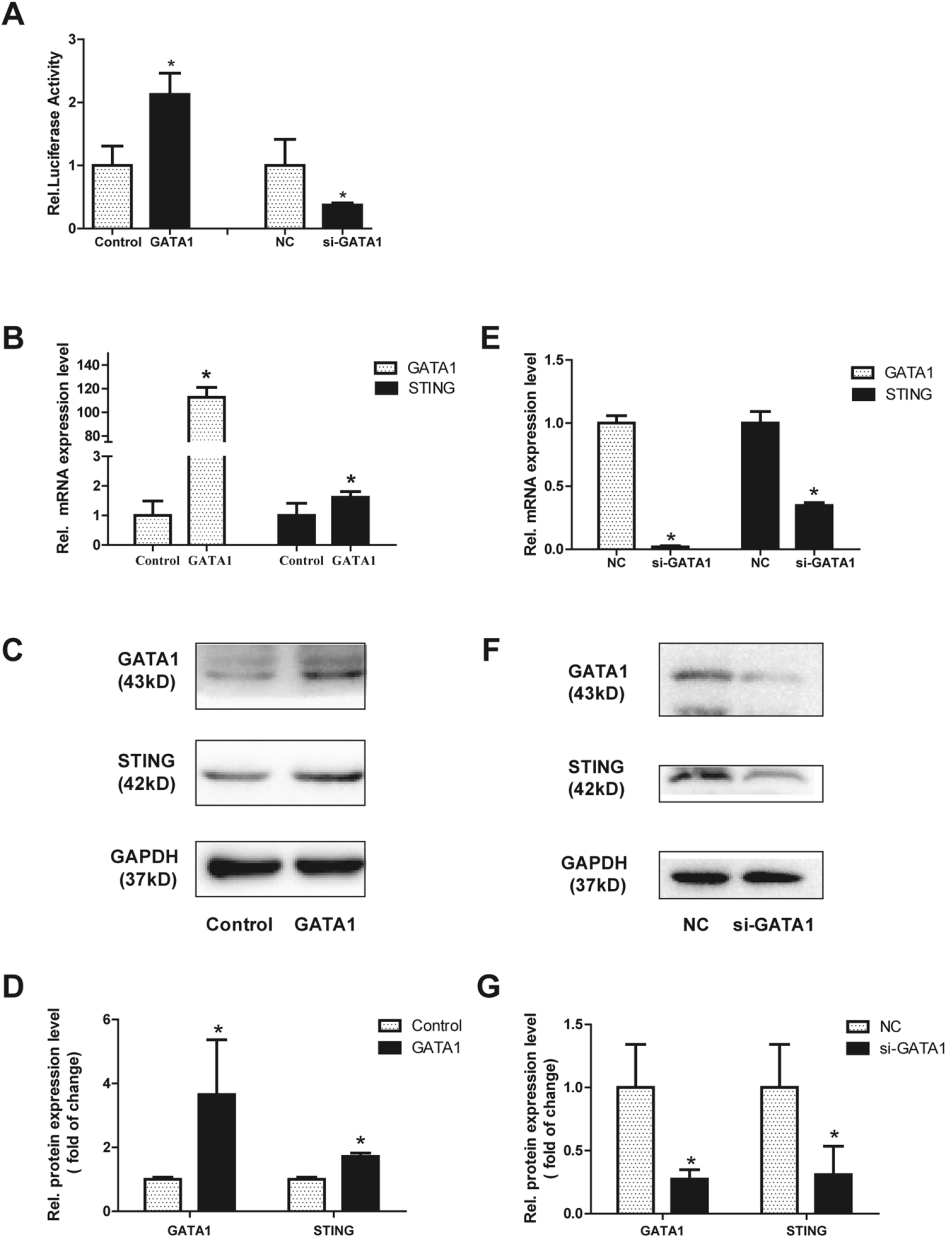
Effect of GATA1 on promoter activity of the mSTING gene. (**A**) Expression of the exogenous GATA1 increased the luciferase reporter gene activity of STING and knockdown of the endogenous GATA1 decreased the promoter activity. NIH3T3 cells were cotransfected with plasmids GATA1 or pcDNA 3.1(+) (200 ng), GATA1 siRNA or negative control siRNA (50 nM) and pSTING-254 (100 ng), pRL-TK (3 ng). Luciferase assays were performed after 24 h. (**B**–**D**) The mRNA and protein levels of mSTING were significantly increased after GATA1 overexpression. qRT-PCR and western blot were performed after GATA1 plasmid or pcDNA 3.1(+) were transiently cotransfected into NIH3T3 cells. The protein expression was quantified to GAPDH. Each bar represented the mean ± SD of three independent experiments (n = 3, *P < 0.05 vs. Control). (**E**–**G**) Knockdown of GATA1 by siRNA (50 nM) reduced the mRNA and protein level of mSTING. The protein expression was quantified to GAPDH. siRNA negative control was set as 1. Each bar represented the mean ± SD of three independent experiments (n = 3, *P < 0.05 vs. NC).

In order to justify the critical regulatory function of the GATA1 site, the overexpression plasmid of GATA1 and pSTING-254 were cotransfected into NIH3T3 cells. Luciferase assays showed that promoter activity of pSTING-254 was increased by about 2 folds in compare with control. In addition, knockdown of the endogenous expression of GATA1 by siRNA significantly decreased pSTING-254 promoter activity by 60% (Fig. 4A). Meanwhile, to examine whether overexpression or knockdown of the GATA1 gene affected the expression of mSTING in living cells, the mRNA and protein levels of mSTING were determined in NIH3T3 cells by qRT-PCR and western blot analysis. The results (Fig. 4B–D) showed that the mRNA and protein levels of mSTING were increased obviously when GATA1 were overexpressed and that siRNA against GATA1 caused almost a half reduction of mSTING mRNA and protein expression compared to negative control (Fig. 4E–G). These results suggested that transcription factor GATA1 positively regulates the mSTING expression in NIH3T3 cells.

### Sp3 instead of Sp1 contributes to the activation of the mSTING promoter

**Figure 5:**
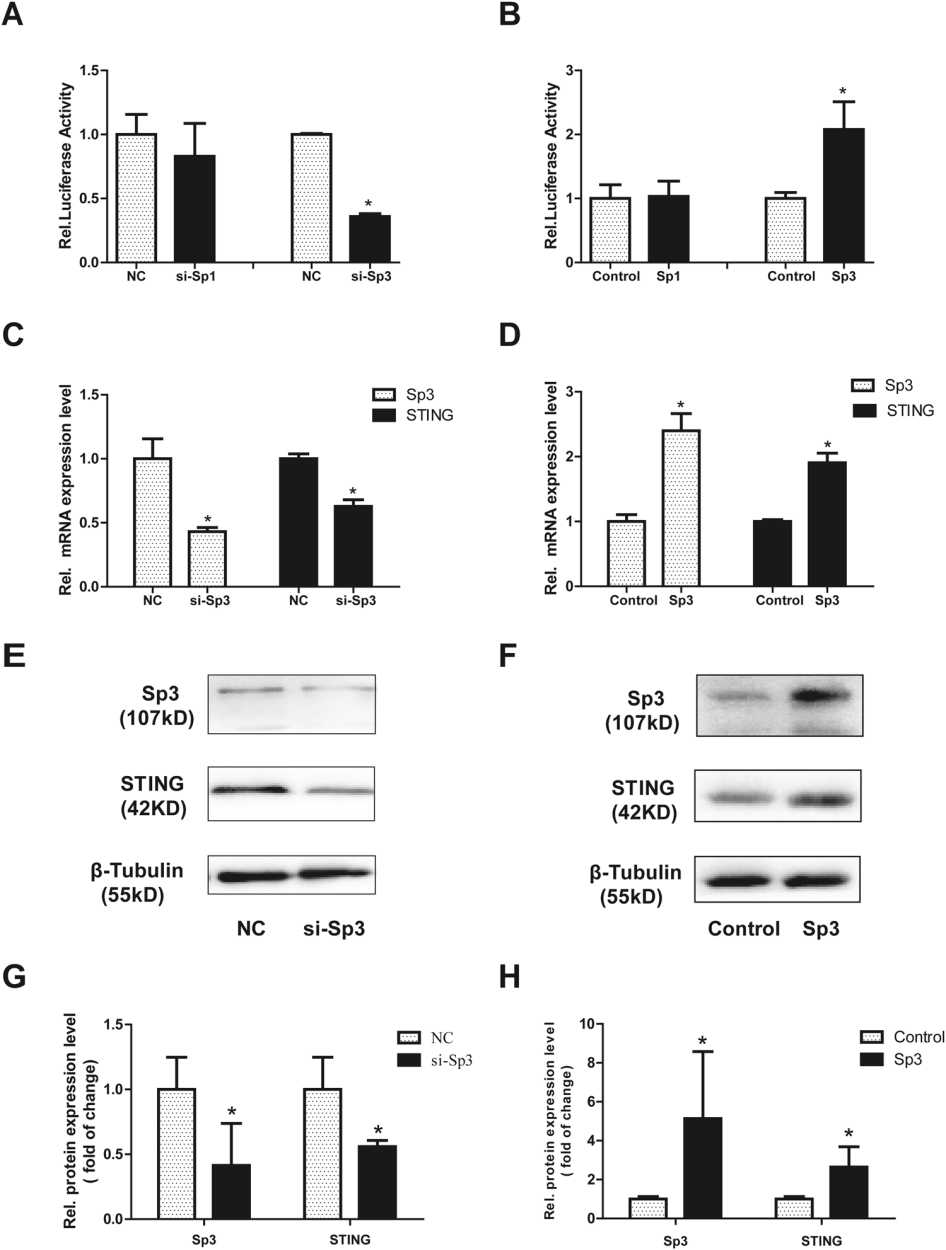
Sp3 but not Sp1 regulated the mSTING proximal promoter activity. (**A**,**B**) Overexpression of Sp1 or knockdown of Sp1 had no effect on the activity of the mSTING promoter. While knockdown of Sp3 by siRNA reduced the mSTING promoter activity to 35%, and overexpression of Sp3 increased the promoter activity by more than 2 folds. NIH3T3 cells were co-transfected with Sp1/Sp3 siRNA (50 nM) or Sp1/Sp3 expressing plasmid (200 ng) and pSTING-254(100 ng) and inner control pRL-TK (3 ng). After 24 h, luciferase activity was measured. (**C**,**E**,**G**) Reduction of endogenous Sp3 expression by siRNA decreased expression of the mSTING gene at mRNA and protein level. qRT-PCR and western blot were performed after siRNA/NC(50 nM) were transiently cotransfected into NIH3T3 cells. siRNA negative control was set as 1. Each bar represented the mean ± SD of three independent experiments. (n = 3, *P < 0.05 vs.NC). (**D**,**F**,**H**) STING protein expression increased by two times under overexpression of Sp3. Sp3 plasmid, pcDNA 3.1(+) (4000 ng) were transiently cotransfected into NIH3T3 cells, and then qRT-PCR and western blot were performed. Each bar represented the mean ± SD of three independent experiments. (n = 3, *P < 0.05 vs. Control).

Mutation analysis showed that the Sp1/Sp3 binding site played an important role in the regulation of the mSTING promoter activity. To further verify the role of Sp1/Sp3 in the regulation of mSTING promoter activity, we cotransfected the reporter plasmids pSTING-254 together with siRNA-Sp1 (si-Sp1), siRNA-Sp3 (si-Sp3) or negative control (NC) into NIH3T3 cells individually. As showed in Fig. 5A, there was no significant change in the luciferase activity of pSTING-254 at the presence of siRNA-Sp1. On the contrary, it showed about 55% reduction of the luciferase activity in the presence of siRNA-Sp3 compared to NC. In accordance with it, overexpression of Sp3 enhanced the promoter activity of pSTING-254 by more than 2 folds, while pSTING-254 failed to response to Sp1 expression vector (Fig. 5B). In addition, to further confirm the critical regulatory function of Sp3 on the expression of mSTING, qRT-PCR and Western blot analysis were performed. siRNA-Sp3 was effective in decreasing the mSTING mRNA and protein levels (Fig. 5C,E,G). And the expression level of mSTING in both mRNA and protein was up-regulated effectively under overexpression of Sp3 (Fig. 5D,F,H). These results indicated that transcription factor Sp3, but not Sp1, contributes to basal transcriptional activity of the mSTING gene.

### GATA1 and Sp3 bind to the mSTING promoter *in vivo* in NIH3T3 cells

**Figure 6:**
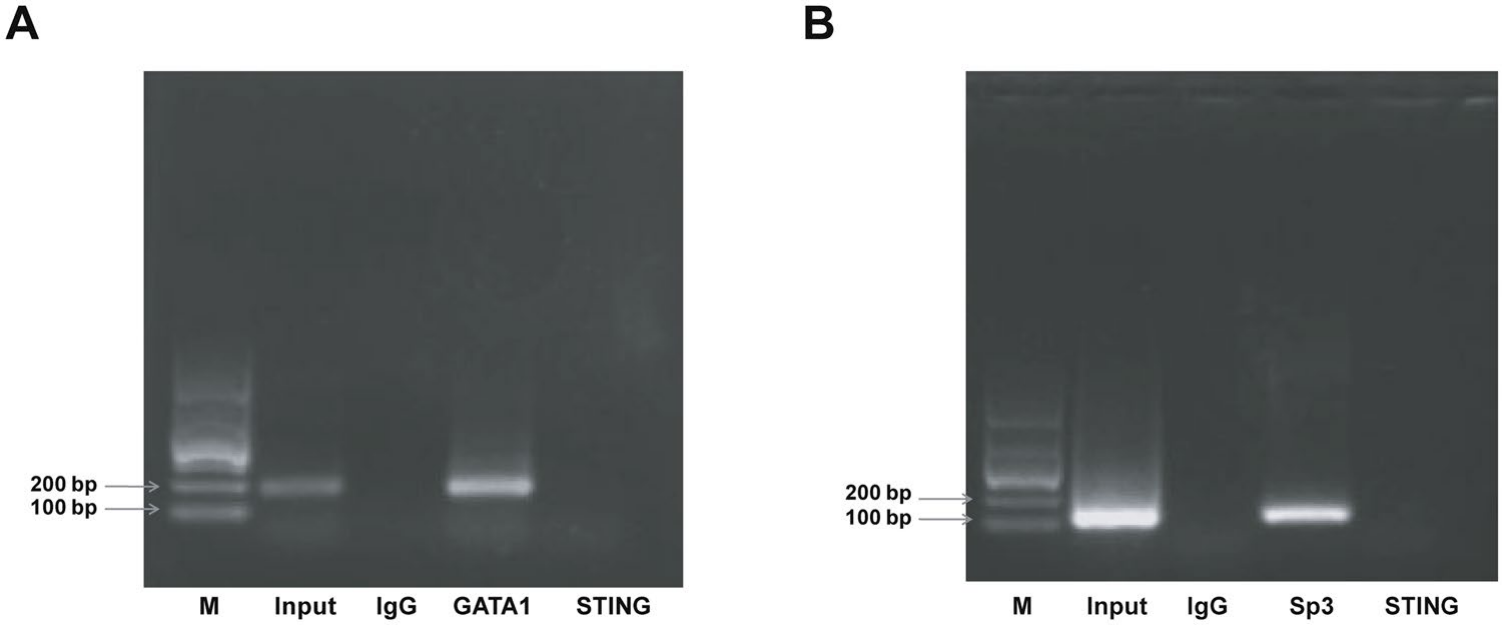
ChIP assay of the mSTING proximal promoter was performed in NIH3T3 cells. The immunoprecipitated chromatin fragments were analyzed by semi-quantitative PCR using primer pairs spanning the putative GATA1 and Sp1 binding site (the target locus) or exon 6 of the mSTING (a non-target locus). (**A**) GATA1 bound to the mSTING promoter *in vivo*. A band of 199 nt containing the GATA1 binding site in the mSTING promoter region was amplified. Lane M: DNA marker 1000. Lane INPUT: PCR product derived by ChIP-GATA1 primers from direct input DNA template without immunoprecipitation. Lane IgG: PCR product derived by ChIP-GATA1 primers from DNA template immunoprecipitated by normal IgG as a negative control. Lane GATA1: PCR product derived by ChIP-GATA1 primers from DNA template immunoprecipitated by anti-GATA1 antibody. Lane STING: PCR product derived by primers flanking exon 6 of the mSTING from DNA template immunoprecipitated by anti-GATA1 antibody. (**B**) Sp3 bound to the mSTING promoter *in vivo*. A band of 107 nt containing Sp3 binding site in the mSTING promoter region was amplified. Lane M: DNA marker 1000. Lane INPUT: PCR product derived by ChIP-Sp3 primers from direct input DNA template without immunoprecipitation. Lane IgG: PCR product derived by ChIP-Sp3 primers from DNA template immunoprecipitated by normal IgG as a negative control. Lane Sp3: PCR product derived by ChIP-Sp3 primers from DNA template immunoprecipitated by anti-Sp3 antibody. Lane STING: PCR product derived by primers flanking exon 6 of them STING from DNA template immunoprecipitated by anti-Sp3 antibody.

To examine whether GATA1 and Sp3 bind to the mSTING promoter region, Chromatin immunoprecipitation (ChIP) assay was performed in NIH3T3 cells. GATA1 and Sp3-associated DNA fragments were immunoprecipated using an antibody that recognized the specific protein subunit, respectively. Normal rabbit IgG was used as a negative control. Then, the DNA precipitated in the complexes was subjected to PCR with primers flanking the region containing the GATA1 or Sp3 binding site. As shown in Fig. 6, GATA1 and Sp3 bound to the mSTING promoter in the motif we had predicted previously *in vivo*, whereas non-specific IgG antibody failed to precipitate protein bound this sequence *in vivo*. Meanwhile, the mSTING primers targeted on the mSTING exon 6 could not amplified any sequences using DNA precipitated by anti-GATA1 or anti-Sp3 protein as the template. These data suggested transcription factor Sp3 and GATA1 are capable of binding to the proximal promoter of the mSTING gene *in vivo*.

## Discussion

STING is a central and multifaceted mediator in innate immune response to cytosolic nucleic especially cytosolic DNA. STING can act both as a direct sensor of CDNs (cyclic dinucleotides) and as an adaptor for the recruitment of downstream signaling components to orchestrate innate immune defenses against various pathogens, abnormal auto-antibodies and cancers. The characterization of transcriptional regulators influencing the STING expression might help to understand these relative diseases’ pathogenesis and progression and provide new target to elucidate therapeutic strategies against these diseases. In our study, we first identified the promoter of the mSTING gene and found the functional proximal minimal promoter of the mSTING gene located from −77 to +177 nt relative to the TSS and the basal transcriptional activity of the mSTING gene is positively regulated by transcription factor GATA1 and Sp3.

The GATA family of transcription factors are highly conserved DNA-binding proteins which have zinc finger DNA domains to recognize the sequences of (A/T)GATA(A/G)^23^. GATA1, the first recognized member of the GATA family (GATA1-6), activates target genes mainly involved in hematopoietic development of erythroid, magakaryocytes, eosinophils, and mast cells^24^. Somatic mutations in the GATA1 gene leading to an N-terminal truncated ‘short’ protein (GATA1s) occurred in nearly all Down syndrome patients with acute megakaryoblastic leukemia (DS-AMKL) and transient myeloproliferative disorder (DS-TMD)^25, 26^. GATA1s mice (knockin mice with exclusively produce GATA1s) revealed critical difference in the expression of type I IFN-response genes compared with wild type mice. Andrew J. Woo’s study showed that loss of type I interferon signaling contributes to GATA1s-induced MkP hyperproliferation, and exogenous IFN-α inhibited hyperproliferation of GATA1s-containing Mks^27^. In our study, we proved that GATA1 positively regulates the expression of the mSTING gene. These results provided some evidences that GATA1 play important roles in immune system.

The transcription factor Sp3 is a constitutively expressed member of the Sp family of transcription factor involved in the expression and regulation of many genes, including housekeeping genes, tissue-specifically expressed genes, viral genes, and cell cycle-regulated genes. Sp1, another member of Sp family, shares over 90% DNA sequence homology in the DNA binding domain. They can recognize and bind to the same DNA element with similar affinity^28, 29^. Usually co-expression of Sp1 and Sp3 compete for the same binding sites *in vivo* and Sp3 acts as an activator or as a repressor depended on Sp1-mediated activations. IRF-3 is expressed ubiquitously in a number of tissues and plays an important role in virus-mediated induction of type I interferon^30^. Xu *et al*. have shown that Sp1 and Sp3 both positively regulate the basal expression of human IRF3 gene by interact with the IRF-3 promoter^31^. In contrast, in normal human prostate epithelial cells, Sp1 positively regulates 15-lipoxygenase 2 (15-LOX2) gene expression, Sp3 reduces the promoter activity of 15-LOX2 gene^32^. Interestingly, in our study, the results showed that Sp1 has no effect on the promoter activity of the mSTING gene whereas Sp3 enhances the mSTING gene expression in NIH3T3 cells. However, the reason why Sp3, instead of Sp1, is the major Sp protein activating the STING promoter region still needs to be confirmed, and whether this regulator mechanism is a cell-type-specific manner still remains unknown.

Although homologues of STING exhibit high sequence similarity in murine and human, some species-specific features lead to interspecies differences in regulation mechanisms and ability to recognize some different ligands. For instance, 10-carboxymethyl-9-acridanone (CMA), a potent type I IFN inducer in mice, directly bound to mSTING protein and triggered a strong antiviral response through the STING-TBK1-IRF3 signaling pathway but failed to activate the hSTING gene because of its inability to bound to the C-terminal ligand binding domain of the hSTING gene^33^. In addition to protein structured differences, the basal transcriptional regulation mechanism of STING was also different between human and murine. We have demonstrated the minimal promoter of human STING is located in the region −124/+1 relative to TSS. Instead of transcription factor GATA1 and Sp3, CREB and c-Myc are involved in the regulation of human STING transcription^34^. All those studies contribute to understand the interspecies differences and may provide some notes when we try to transform achievements from animal experiments to clinical experiments.

In conclusion, this study showed that the mSTING proximal promoter is located within the region of −77 to +177 nt relative to TSS and that transcription factor GATA1 and Sp3 sustain basal transcription activity of the mSTING gene.

## Materials and Methods

### Cell culture

Mouse embryonic fibroblast cells (NIH3T3) and human embryonic kidney 293 (HEK293) cells were obtained from the American Type Culture Collection (ATCC) (http://www.atcc.org) and cultured in high glucose Dulbecco’s modified Eagle’s medium (DMEM) supplemented with 10% (v/v) heat inactivated fetal bovine serum (FBS), 100 U/ml penicillin and 100 μg/ml streptomycin. Cells were incubated at 37 °C in 5% carbon dioxide with humidified atmosphere.

### Cloning of the 5′upstream nucleotide sequences of mSTING gene

A 1005 nt of 5′-flanking region of the mSTING gene was cloning as a potential promoter region using murine genomic DNA as the template. TSS was set according to the first nucleoside of the published mSTING gene sequences (No:NC_000084.6, Gene ID: 72512). Primers were designed by Primer Primier 5.0 software, in which *KpnI* restriction sites (underlined) and protective bases were introduced to the 5′ end of the forward primer, and *HindIII* restriction site (underlined) and protective bases were added to the 5′ end of the reverse primer. The PCR was carried out using F1 and R1 as primers (Table 1) and EXtaq (Takara, Japan) as polymerase. The reaction was incubated at 94 °C for 5 min, repeated 30 cycles with 94 °C for 30 s, 60 °C for 30 s, and 72 °C for 2 min, followed by 10 min at 72 °C.

**Table 1:** Sequences of oligonucleotides used to clone the mSTING gene promoters and site-directed mutagenesis.

Names of plasmids	Primer sequence (5′-3′)	
	Forward	Reverse
pSTING-1005	F1: CGG*GGTACC*TCATGCTGTCATCCACCCAGTTA	R1: CCC*AAGCTT*GCAGGACTCCATACAAGGACCAA
pSTING-771	F2: CGG*GGTACC*CCCGGGCCTCAGCAGCTAGCTT	R1: CCC*AAGCTT*GCAGGACTCCATACAAGGACCAA
pSTING-558	F3: CGG*GGTACC* *GCCAGATGGCTAGCAGGGAAGA*	R1: CCC*AAGCTT*GCAGGACTCCATACAAGGACCAA
pSTING-254	F4: CGG*GGTACC*CAACCATCCTGAGACTGGGA	R1: CCC*AAGCTT*GCAGGACTCCATACAAGGACCAA
pSTING-206	F5: CGG*GGTACC*AGAGTGCTCTCCCCCCTC	R1: CCC*AAGCTT*GCAGGACTCCATACAAGGACCAA
pSTING-164	F6: CGG*GGTACC*TTCCTTGCTCAGATTTCAGG	R1: CCC*AAGCTT*GCAGGACTCCATACAAGGACCAA
pSTING-125	F7: CGG*GGTACC*TCTCAATCTCTCCTGTCTAACC	R1: CCC*AAGCTT*GCAGGACTCCATACAAGGACCAA
GATA1-mut	F8: TCCCAGTCTCAG*ACC*GGTTGGGTACC	R8: AATTAGAGGCGTGGTTATTTCCCCGAAAAG
IK2-mut	F9: CGCCTCTAATT*CTT*CAGTCTCAGGAT	R9: TGGTTATTTCCCCGAAAAGAGTGCTCTC
Sp-mut	F10: GGGAAATTAGA*AAA*GTGGTTATTTC	R10: AGTCTCAGGATGGTTGGGTAC
STAT-mut	F11: GCGTGGTTAT*GGTT*CCGAAAAGAG	R11: CTCTAATTTCCCAGTCTCAGGATGGTTG

Primers for cloning promoter of the mSTING gene: *KpnI* restriction site (underlined and italics) and protective bases were introduced to the 5′ end of the forward primers, and *HindIII* restriction site (underlined and italics) and protective bases were added to the 5′ end of the reverse primers. Primers for site-directed mutagenesis: the bold italics indicated the mutation sites.

### Construction of the mSTING gene promoter recombinant plasmid

The PCR products were digested with *Kpn* *I* and *Hind* *III* (Thermo Fisher Scientific, USA) and then subcloned to promoter-less reporter gene vector pGL3-Basic prepared by *Kpn* *I* and *Hind* *III* double restrictive digestion. The recombinant plasmid was tested and sequenced, and the positive cloned plasmid was named as pSTING-1005. Similarly, the 5′ deletion clones were amplified with PCR by the reverse primerR1, which paired with the forward primers listed in Table 1. The deletion fragments with different length were subcloned into pGL3-Basic vector. The promoter recombinant plasmids were confirmed by sequencing and named as pSTING-771(−594/+177), pSTING-558(−381/+177), pSTING-254(−77/+177), pSTING-206(−29/+177), pSTING-164(+13/+177) and pSTING-125(+52/+177), respectively.

### Plasmids and siRNAs

The expression plasmids of GATA1, pcDNA empty vector were stored by our group. The expression plasmids Sp1 and Sp3 were kindly provided by Dr. Guntram Suske. GATA1 siRNA, Sp1 siRNA, and Sp3 siRNA and the negative control (NC) were designed and synthesized in Genepharma Company (Shanghai, China). Sequences targeted in the GATA1, Sp1 and Sp3 mRNA, as well as the negative control sequence were below: si-Sp1: 5′-UUGAGUCACCCAAUGAGAA-3′; si-Sp3: 5′-UCAUUCCUGGCUCUAAUCAA-3′; si-GATA1: 5′-UGGCGGAGGGACAGGACA-3′; NC: 5′-UUCUCCGAACGUGUCACGUTT-3′.

### Site-directed mutagenesis

The promoter region (−77/+52 nt) of the mSTING gene were predicted by online software TFSEARCH ver 1.3 (http://mbs.cbrc.jp/research/db/TFSEARCH.html). Oligonucleotides with site-specific mutations at the critical nucleotides necessary for transcription factor (Table 1) were generated using the QuickChange Site-directed Mutagenesis kit (TAKARA, Japan) on the backbone of wild-type promoter pSTING-254 according to the manufacture’s protocol. And the mutations were confirmed by sequencing. The site-special mutagenized plasmids were named as GATA1-mut, IK2-mut, Sp-mut, STAT-mut and GATA1/Sp-mut according to the binding sites.

### Transient transfections and luciferase assays

Transient transfections were carried out in NIH3T3 or HEK293 cells using Lipofectamine^TM^2000 (Invitrogen, USA) according to the manufacturer’s suggestion. Cells were vaccinated in a 96-well culture plates and when these cells had grown to70–80% before transfection. For luciferase assays, 200 ng of each of the luciferase recombinant plasmids was cotransfected into NIH3T3 cells with 2 ng of control pRL-TK plasmids as an internal control. For overexpression or knockdown by siRNA, expression plasmid (200 ng) or siRNA (50 nM) of GATA1, Sp1 or Sp3 was individually cotransfected into NIH3T3 cells with 100 ng of pSTING-254 luciferase recombinant plasmids together with 3 ng of control pRL-TK plasmids as an internal control. Cells were harvested after 24 hours. Quantification of firefly and Renilla luciferase activities of at least three independent transfections was measured with the Dual Reporter assay system (Promega, USA) using FB12 luminometer (Berthold, Germany). The relative luciferase activities (RLA) were calculated by normalizing the fluorescence luciferase with internal standard Renilla luciferase.

### RNA Extraction and quantitative real-time PCR

Total RNA was extracted from NIH3T3 cells using TRIzol reagent (Invirtrogen, USA) followed by chloroform-isopropanol extraction and ethanol precipitation. Then total RNA was used as the first strand to synthesis cDNA by reverse transcription. Subsequently, the quantification of gene transcripts was performed by real-time PCR using SYBR Green I dye (TAKARA, Japan) and ABI 7300 real-time PCR system (Applied Biosystems Inc.). The forward and reverse primer sequences were designed using Primer Primier 5.0 software and listed in Table 2. The β-actin gene was used as an internal standard. The specificity of amplification was assessed by melting curve analysis for each sample. The ΔΔCt method was used to transform Ct values into relative quantities (mean ± standard deviation). Changes were expressed as a percentage of the controls.

**Table 2:** Sequences of oligonucleotides used in RT-PCR and ChIP assay.

Gene	Forward(5′-3′)	Reverse(5′-3′)
β-actin	GTGACGTTGACATCCGTAAAGA	GCCGGACTCATCGTACTCC
STING	TGGCCTTCTGGTCCTCTATAA	CTCGTAGACGCTGTTGGAATAA
mGATA1	TGTCCTCACCATCAGATTCCA	TCCCTCCATACTGTTGAGCAG
hSp1	GGCTGTGGGAAAGTGTATGG	GGCAAATTTCTTCTCACCTGTG
mSp1	GGCTGCCCATTTGTACTCATTTAC	CCGAAGGGTGCCTGTTAGG
hSp3	TTCAGGGAGTTGCAATTGGTG	TTCTGTGCCTGTGTCTCTTCA
mSp3	AAGCTGGAAGAGATGATGCCT	TGACACAGACCCCTGTTGAA
ChIP-GATA1	AGAAGCCTTTGGCTATCTGG	GAGAGATTGAGATGAACAGC
ChIP-Sp3	ACTGGGAAATTAGAGGCGTG	GAGAGATTGAGATGAACAGC

### Western blot analysis

The whole-cell extracts were prepared by washing cells twice with ice-cold 1× PBS and lysed in lysis buffer with the addition of Protease Inhibitor Cocktail, Phosphatase Inhibitor Cocktails and PMSF (Kangchen bio-tech, China) for 30 min at 4 °C on a rocking platform before scraping and transferring to tubes. The lysates were cleared by centrifugation at 13,000 *g* for 20 min at 4 °C, and protein quantification was performed with the BCA protein assay kit (Beyotime, China). For western blotting, 20 μg total protein were prepared with 5× SDS sample buffer and resolved by 10% SDS-PAGE gel and transferred onto polyvinylidene difluoride (PVDF) membrane (ImmobilinP, Millipore) using standard procedures. After blocking in 5% skim milk in Tris-buffered saline–Tween (TBST), incubation for overnight at 4 °C with primary antibodies against GAPDH, β-Tubulin, Sp1, Sp3 (Santa Cruz, USA), GATA1 and STING (Abcam, UK) and then with HRP-conjugated secondary antibodies (Abcam, UK) was performed. Subsequently, Chemoluminescense signals of membranes were quantified with ECL reagent (Pierce, USA) and visualized by enhanced chemiluminescence (Cell Signaling Technology, Inc). Anti-GAPDH or β-Tubulin was used as loading control.

### Chromatin immunoprecipitation (ChIP) assay

ChIP assays were performed with the Magna ChIP™ kit (Millipore, USA) following the manufacturer’s instructions. A total of 1 × 10^7^ cells were needed for ChIP assay. NIH3T3 cells were fixed with 1% formaldehyde for 10 min at room temperature. After fixation, cells were harvested by centrifugation at 4 °C. The cell pellets were resuspended in nuclear lysis buffer and sonicated using six 15 sec pulses with 50 second rest in between pulses at 5% of maximum output strength on a Sonicator Ultrasonic Processor (Qsonica, LLC). The chromatin was immunoprecipitated with anti-IgG (Millipore USA), anti-Sp3 and anti-GATA1 antibody. After reverse cross-linking and DNA purification, DNA from input or immunoprecipitated samples were assayed by PCR with the appropriate promoter primers listed in Table 2. ChIP-GATA1 primers were designed to amplify the region including predicted GATA1 binding sites, ChIP-Sp3 to amplify the region including putative Sp3 binding sites, and STING primers to amplify exon 6 of the mSTING gene without GATA1 or Sp3 binding site as a non-target locus. The PCR amplified products were separated by 1% agarose gel electrophoresis with ethidium bromide, and the PCR signals were analyzed by quantification.

### Statistical Analysis

Data were presented as means ± SD and represented at least three independent experiments, and differences between groups were analyzed by ANOVA using SPSS 18.1 software. For all statistical comparisons, a p < 0.05 was considered statistically significant.
